# Evaluating the performance of different Bayesian count models in modelling childhood vaccine uptake among children aged 12–23 months in Nigeria

**DOI:** 10.1186/s12889-023-16155-z

**Published:** 2023-06-21

**Authors:** A. F. Fagbamigbe, T. V. Lawal, K. A. Atoloye

**Affiliations:** Department of Epidemiology and Medical Statistics, Faculty of Public Health, University of Ibadan, Ibadan, Nigeria

**Keywords:** Poisson, Negative binomial, Zero-inflated Poisson, Zero-inflated negative binomial, Child Vaccination, Immunization, Nigeria

## Abstract

**Background:**

Choosing appropriate models for count health outcomes remains a challenge to public health researchers and the validity of the findings thereof. For count data, the mean–variance relationship and proportion of zeros is a major determinant of model choice. This study aims to compare and identify the best Bayesian count modelling technique for the number of childhood vaccine uptake in Nigeria.

**Methods:**

We explored the performances of Poisson, negative binomial and their zero-inflated forms in the Bayesian framework using cross-sectional data pooled from the Nigeria Demographic and Health Survey conducted between 2003 and 2018. In multivariable analysis, these Bayesian models were used to identify factors associated with the number of vaccine uptake among children. Model selection was based on the -2 Log-Likelihood (-2 Log LL), Leave-One-Out Cross-Validation Information Criterion (LOOIC) and Watanabe-Akaike/Widely Applicable Information Criterion (WAIC).

**Results:**

Exploratory analysis showed the presence of excess zeros and overdispersion with a mean of 4.36 and a variance of 12.86. Observably, there was a significant increase in vaccine uptake over time. Significant factors included the mother’s age, level of education, religion, occupation, desire for last-child, place of delivery, exposure to media, birth order of the child, wealth status, number of antenatal care visits, postnatal attendance, healthcare decision maker, community poverty, community illiteracy, community unemployment, rural proportion and number of health facilities per 100,000. The zero-inflated negative binomial model was best fit with -2Log LL of -27171.47, LOOIC of 54464.2, and WAIC of 54588.0.

**Conclusion:**

The Bayesian zero-inflated negative binomial model was most appropriate to identify factors associated with the number of childhood vaccines received in Nigeria due to the presence of excess zeros and overdispersion. Improving vaccine uptake by addressing the associated risk factors should be promptly embraced.

## Introduction

The Generalized Linear Model (GLM) is a flexible modelling framework to explore distributional forms other than the normal distribution through an arbitrary choice of link functions [1, 2]. The outcome variable, when normally distributed, can be applied to classical models which include analysis of variance and ordinary least squares regression but other distributional forms in the exponential family, such as binomial, Poisson, negative binomial or gamma distribution can be applied otherwise [1, 3, 4]. Moreover, the parameter of interest cannot be modelled as a line, as it is certain to yield negative values for the outcome variable [3, 4].

Since vaccination coverage is a random count event, and typically independent, the use of traditional statistical techniques, particularly the ordinary least squares and the logistics regression will be inappropriate as their assumptions will be violated and thereby bias the resulting estimates. Therefore, the Poisson distribution is a very intuitive means to describe the randomness of count outcomes [2, 4]. The Poisson regression, as the default count model, models the noisy output of a counting function, y, as a Poisson random variable, with a log-mean parameter that is presented as a linear function [3]. For count data, the mean–variance relationship and proportion of zeros are a major determinants of the choice of model to be used, which ranges from the Poisson and negative binomial to the zero-inflated Poisson and zero-inflated negative binomial to the hurdle models [2, 5].

The Poisson regression has the advantage of fitting nonlinear models as against the conventional linear regression models, including situations that involve the number (count) of occurrences of an event. This regression model has been used and recommended earlier [3, 4, 6]. However, these studies focused on the frequentist approach without any consideration for the Bayesian techniques. More so, the Poisson model might be inappropriate for over-dispersed data[4, 5]. The Negative Binomial regression model is more flexible and is used to model count data with overdispersion, although the Negative Binomial can also be inappropriate when explaining overdispersion in that the mean also implies that the variability of the outcome variable having the values of the same covariates is equal to the mean [1, 2, 4, 7].

To understand the underlying two types (structural/true and random/false zeros) of zero inflation in count data, there approaches that a researcher employs to model such zero-inflation [1]. In particular, the Zero-inflated Poisson and Zero-inflated Negative Binomial are applicable when Poisson and Negative Binomial are inappropriate in handling overdispersion due to a high number of zeros [4, 7]. The Poisson hurdle model is also an alternative that can model all zeros as one part and a zero-truncated part for all the non-zero observations [1–3]. Unlike the zero-inflated Poisson, the hurdle model assumes that all zero observations come from the same group. An advantage of the Zero-Inflated Negative Binomial model is that it is more robust in the face of overdispersion due to a high number of zeros, and incorporates an additional dispersion parameter that factors in over-dispersion that is generated from positive values [1, 4], and reduce bias that results from non-normality of the outcome variable.

Bayesian inference (which focuses on infusing prior knowledge into models by applying probability theory) can be applied through the Markov Chain Monte Carlo (MCMC) simulation to generate random values with Hamiltonian Monte Carlo (HMC) algorithm. The Bayesian specification is more flexible for parameter estimation than the traditional models [4, 8].

Across the literature, researchers have also identified some factors that may affect vaccination uptake among children. Some of the factors included demographic and socio-economic factors including child-specific, parental and household characteristics have been identified as important predictors of child vaccination uptake [9–12]. Similarly, some child-specific characteristics such as birth order, place of birth of child, and age of the child have been found to influence vaccination uptake [10, 12].

While previous studies have extensively explored count models for analyzing childhood vaccine uptake, there is a lack of research that systematically compares the performance of different count models, particularly from a Bayesian perspective, and identifies the most suitable model for modeling childhood vaccine uptake in Nigeria. Additionally, there is limited understanding of the factors that may influence childhood vaccine uptake in the Nigerian context. Therefore, there is a need for a comprehensive study that not only compares the performance of count models but also investigates the key factors affecting childhood vaccine uptake in Nigeria. This research gap hinders the ability of public health researchers to make informed decisions regarding the selection of an appropriate count model for analyzing vaccine uptake and understanding the factors that contribute to it. The study, therefore, compared the performance of count models and identify the best Bayesian count model for the number of childhood vaccine uptake in Nigeria. This paper will be useful for public health researchers in the choice of an appropriate model of count health outcomes, and also help identify some factors that may influence such.

## Methods

### Study design

This is a methodological paper that details the performances of different Bayesian count models. We started with the review of the models, described the data we used and applied the models to the multi-year childhood vaccination data in Nigeria.

### The models

Four count regression modelling techniques were assessed and implemented in the Bayesian multilevel framework under the generalized linear mixed model (GLMM) with both fixed and random effects. The 3 levels were defined for children i, who took vaccination (at level 1), from a community j (at level 2) and living in a state k (at level 3). The second and third levels were particularly useful to account for the inherent spatial and temporal autocorrelation in childhood vaccination uptake.

### Models fitted to the data

**Table:** 

Distribution	Regression	Notes *
${y}_{i,j,k} \sim Poisson\left({\lambda }_{i,j,k}\right)$	$log\left({\lambda }_{i,j,k}\right)={\beta }_{0}+{\beta }_{i}{X}_{i}+{e}_{i,j,k}$	1
${y}_{i,j,k} \sim NB\left({\lambda }_{i,j,k}\right)$	$log\left({\lambda }_{i,j,k}\right)={\beta }_{0}+{\beta }_{i}{X}_{i}+{e}_{i,j,k}$	2
${y}_{i,j,k} \sim ZIP\left(\theta , {\lambda }_{i,j,k}\right)$	${\upeta }_{ijk}={\beta }_{0}+{\beta }_{i}{X}_{i,j,k}+{(e}_{i,j,k }+{r}_{i,j,k})$	1 2 3 4
${y}_{i,j,k} \sim ZINB\left(\theta , {\lambda }_{i,j,k}\right)$	${\upeta }_{ijk}={\beta }_{0}+{\beta }_{i}{X}_{i}+{(e}_{i,j,k }/{r}_{i,j,k })$	1 2 3 4

where 1 – vector ${e}_{i,j,k}$ denotes the random effects components for Poisson or NB; 2 – ${X}_{i}$ has the full rank p and q for the logistic and the Poisson/NB components; 3 – ${\upeta }_{ijk}$ is the link function with both log and logit; 4 – ${r}_{i,j,k}$ is the random effects component for the logistics part of the zero-inflated models

For each of the distributions specified above, their models can be written as:

- (i) Poisson:
  $$\mathrm{Pr}\left({Y}_{i}={y}_{i}\right)=\frac{{e}^{-\mu }{\mu }^{{y}_{i}}}{{y}_{i}!}, {y}_{i}=\mathrm{0,1},2,\dots$$
- (ii) Negative Binomial:
  $$\mathrm{Pr}\left({Y}_{i}={y}_{i}\right)=\frac{\Gamma (k+{y}_{i})}{\Gamma (k){y}_{i}}{{\left(\frac{\mu }{k+\mu }\right)}^{k}\left(\frac{\mu }{k+\mu }\right)}^{{y}_{i}}$$

where μ is the mean, and k represents the dispersion parameter. The variance of the distribution is $\left(\mu +\frac{{\mu }^{2}}{k}\right)$, which implies that decreasing values of k will lead to increasing levels of dispersion; and as k increases towards the positive infinity, a Poisson distribution is obtained [1]. Therefore, unlike the Poisson model, the negative binomial model can capture the level of over-dispersion, but it did not solve the problem of overdispersion [1, 5].

- (iii) Zero-inflated Poisson:
  $$\mathrm{Pr}\left({Y}_{i}={y}_{i}\right)=\left\{\pi \begin{array}{c}(1-\pi )+\pi {e}^{-\mu }, for {y}_{i}=0\\ \frac{{e}^{-\mu }{\mu }^{{y}_{i}}}{y!\left(1-{e}^{-\mu }\right)}, for {y}_{i}=1, 2, \dots \end{array}\right.$$
- (iv) Zero-inflated Negative Binomial
  $$\mathrm{Pr}\left({Y}_{i}={y}_{i}\right)=\left\{\begin{array}{c}{\pi }_{i}+(1-{\pi }_{i}){(1+k{\mu }_{i})}^{-\frac{1}{k}}, for {y}_{i}=0\\ (1-{\pi }_{i}) \frac{\Gamma \left({y}_{i}+\frac{1}{k}\right){\left(k{\mu }_{i}\right)}^{{y}_{i}}}{\Gamma \left({y}_{i}+1\right)\Gamma \left(\frac{1}{k}\right){(1+k{\mu }_{i})}^{{y}_{i}+\frac{1}{k}}}, for {y}_{i}=1, 2, \dots \end{array}\right.$$

### Model selection

Model selection and comparison were done using -2 Log Likelihood (-2 Log LL), Leave-One Out Cross-Validation (LOOIC) and the Widely Applicable or Watanabe Akaike Information Criterion (WAIC). The modelling technique with the lowest of all these information criteria was selected as the most robust for our analysis.

### Implementation of the models

All models considered in this study were executed using the Bayesian multilevel regression modelling technique in Stan (Sampling Through Adaptive Community), a probabilistic programming language, embedded in R. Stan, which was implemented in the ‘brms’ package, is a C +  + package designed for performing complex hierarchical Bayesian inference [13, 14].

Chain convergence was achieved using weakly informative priors – derived from the data. The number of initial iterations (including warmup) was set to 15000, chain at 4 and thin (how frequently the parameter estimates are refreshed during iterations) at 50.

### Data

The data is from the multi-year cross-sectional nationally representative population-based household Nigeria Demographic and Health Survey (NDHS). Data were pooled from the successive NDHS conducted in 2003, 2008, 2013 and 20018 in Nigeria. The NDHS used a multistage stratified sampling design (from clusters known as primary sampling units (PSUs) to the selection of the households for all the years. We computed survey-women weights (SWW) to the analysis to reflect the differences in population sizes of the women in each survey. The SWW is the product of DHS-provided weights and survey-specific weights (SSW). SSW was computed as the number of sampled women aged 15–49 years divided by the population of women aged 15–49 years during each survey. However, since we were not interested in a pooled estimate, but rather individual survey estimates, we don’t need to apply further sampling besides the sampling weights already provided via v005 in the original dataset.

#### Dependent variable

The outcome variable is the number of vaccines taken by the children. The mothers provided information on whether a child received a specific vaccine or not. The data on each of the 9 vaccines were then merged to obtain the number of vaccines a child took. The vaccines are one dose of Bacillus-Calmette-Guérin (BCG); three doses of Diphtheria-Pertusis-Tetanus (DPT); three doses of Polio and one dose of Measles.

#### Independent variables

Explanatory Variables were selected based on findings in the literature [9, 10, 12, 15] and the availability of data at individual, community and state levels. Three levels of explanatory variables were used for the hierarchical nature of the study:

Individual variables include socio-demographic characteristics of the child and mother’s age in years, mother’s educational level, mother’s religion, mother’s occupation, desire for the child, place of delivery, exposure to media, birth order, wealth status, number of antenatal care visits and postnatal care attendance within 2 months of delivery, sex of the child, and healthcare decision-maker, were included in the analysis. Exposure to media, in this study, was defined as the mother’s access to information through any newspapers/magazines, radio or television (i.e., if the mother reads or watches any, at least once a week).

Community-level variables such as place of residence, community poverty rate, illiteracy rate and unemployment, and State level variables such as the proportion of the rural population, and health facilities per 10,000 population were included in the model.

## Results

**Fig. 1:**
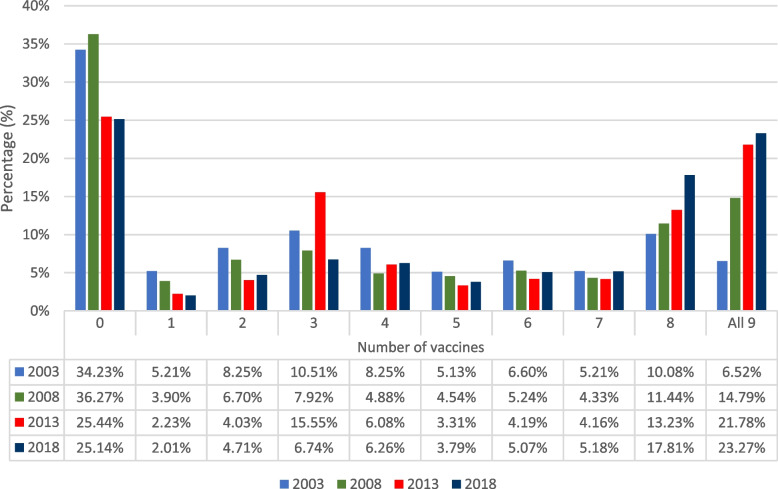
Percentage distribution of number of vaccines taken between 2003 and 2018

The distribution of the number of vaccine uptake among the children was presented in Fig. 1, and disaggregated by survey years. A consistently high number of zeros was observed in the data, across survey years – this confirms that the structure of the data on the number of vaccine uptake in Nigeria has a high number of zeros, and is suggestive of overdispersion.

Table 1 presents the comparison of the model parameters and estimates. The mean number of vaccines taken was less than the variance; this suggests that the Poisson regression may be unsuitable. Nonetheless, further statistical tests were conducted to determine the optimal model.

**Table 1:** Comparison and selection of the models

	**Mean**	**Median**	**Variance**
**Number of Vaccination**			
** 2003**	3.27	3	9.89
** 2008**	3.70	3	12.58
** 2013**	4.54	4	12.44
** 2018**	4.94	6	12.94
**Overall**	4.36	4	12.82
	**-2 Log Likelihood**	**LOOIC**	**WAIC**
** Poisson**	-31384.02	63586.2	64503.7
** Negative Binomial**	-30274.49	60769.7	60995.8
** Zero Inflated Poisson**	-27235.46	54601.6	54735.4
** Zero Inflated Negative Binomial**	-27171.47	54464.2	54588.0

The -2 Log LL, LOOIC and WAIC were used as further tests of the goodness of fit for model comparison and selection. Although the Zero-Inflated Poisson had similar estimates and model fit statistics with the Zero-Inflated Negative Binomial, the Zero-Inflated Negative Binomial had the smallest values for all -2 Log LL, LOOIC and WAIC; which makes it a more applicable model to fit the data on the number of vaccines uptake better.

Furthermore, in Table 2, the estimates of each of the count models employed in this study were presented, alongside the standard error. It was observed that the estimates of the Poisson and Negative binomial regression were similar, although little differences were seen in the estimates. The same similarities were observed in the Zero-inflated Poisson and Zero-inflated Negative Binomial estimates.

**Table 2:** Count modelling technique and estimate comparison

	**Poisson** **[β (SE)]**	**Negative Binomial** **[β (SE]**	**Zero-inflated Poisson** **[β (SE)]**	**Zero-inflated Negative Binomial** **[β (SE)]**
**CONTROL VARIABLE**				
**Year**				
2003	Reference			
2008	22.63 (4.51) *	12.53 (3.86) *	12.20 (1.99) *	12.24 (8.86) *
2013	22.41 (4.48) *	12.32 (3.79) *	12.35 (2.00) *	12.39 (8.85) *
2018	22.32 (4.47) *	12.22 (3.82) *	12.38 (2.02) *	12.44 (8.86) *
**FIXED EFFECTS**				
**Level 1 (Individual Level Characteristics)**				
**Age**				
15 – 24 years	Reference			
25 – 34 years	0.08 (0.01) *	0.06 (0.02) *	0.06 (0.01) *	0.04 (0.01) *
35 – 49 years	0.09 (0.01) *	0.08 (0.02) *	0.09 (0.01) *	0.07 (0.02) *
**Level of Education**				
No formal education	Reference			
Primary education	0.17 (0.03) *	0.19 (0.00) *	0.10 (0.02) *	0.10 (0.01) *
Secondary/Tertiary education	0.24 (0.04) *	0.28 (0.02) *	0.14 (0.02) *	0.14 (0.01) *
**Religion**				
Christian	Reference			
Islam	-0.10 (0.01) *	-0.14 (0.03) *	-0.08 (0.02) *	-0.07 (0.02) *
Others	-0.27 (0.05) *	-0.31 (0.08) *	-0.22 (0.04) *	-0.16 (0.02) *
**Occupation**				
Unemployed/Unskilled manual	Reference			
Employed	0.10 (0.01) *	0.11 (0.05) *	0.04 (0.02) *	0.04 (0.02) *
Self employed	0.10 (0.01) *	0.13 (0.02) *	0.05 (0.02) *	0.05 (0.01) *
Others	0.08 (0.01) *	0.11 (0.05) *	0.03 (0.02) *	0.03 (0.02) *
**Wanted last child**				
Wanted then	Reference			
Wanted later	-0.00 (0.03)	-0.02 (0.02)	-0.04 (0.02) *	-0.03 (0.02) *
Wanted no more	-0.02 (0.04)	-0.04 (0.06)	-0.03 (0.02) *	-0.01 (0.02)
**Place of delivery**				
Home or elsewhere	Reference			
Hospital/health center	0.07 (0.03) *	0.08 (0.01) *	0.03 (0.01) *	0.04 (0.01) *
**Exposure to media**				
Not exposed to media	Reference			
Exposed to media	0.05 (0.00) *	0.04 (0.02) *	0.01 (0.01)	0.02 (0.02) *
**Birth order of child**				
First	Reference			
Second	-0.01 (0.01)	0.02 (0.02)	-0.03 (0.01) *	-0.02 (0.02)
Third	-0.00 (0.01)	0.03 (0.03) *	-0.03 (0.01) *	-0.02 (0.02) *
Fourth and above	-0.04 (0.02) *	0.01 (0.02)	-0.05 (0.01) *	-0.02 (0.03)
**Wealth Status**				
Bottom 33%	Reference			
Average	0.07 (0.01) *	0.09 (0.01) *	0.07 (0.01) *	0.07 (0.00) *
Top 33%	0.17 (0.02) *	0.22 (0.02) *	0.14 (0.05) *	0.12 (0.02) *
**Number of ANC visits**				
No visit	Reference			
1 – 3 visits	0.42 (0.02) *	0.44 (0.03) *	0.28 (0.02) *	0.25 (0.01) *
4 – 7 visits	0.52 (0.01) *	0.57 (0.01) *	0.34 (0.03) *	0.32 (0.01) *
8 or more visits	0.50 (0.01) *	0.54 (0.02) *	0.31 (0.03) *	0.30 (0.00) *
**Sex of the child**				
Male	Reference			
Female	0.02 (0.01) *	0.02 (0.02) *	-0.01 (0.01)	0.00 (0.01)
**Postnatal Care attendance within 2months**^**7**^				
No	Reference			
Yes	0.19 (0.00) *	0.23 (0.02) *	0.08 (0.01) *	0.07 (0.01) *
**Healthcare decision maker**				
Self	Reference			
Husband alone	0.01 (0.02)	-0.05 (0.01) *	-0.00 (0.00)	0.00 (0.01)
Joint	0.03 (0.01) *	-0.02 (0.01) *	0.01 (0.02)	0.01 (0.00) *
Other	-0.18 (0.03) *	-0.24 (0.13) *	-0.15 (0.01) *	-0.10 (0.08) *
**Level 2 (Community Level Characteristics)**				
**Place of residence**				
Urban	Reference			
Rural	0.02 (0.02)	0.03 (0.03) *	0.00 (0.03)	-0.00 (0.01)
**Community poverty rate**				
Low	Reference			
Average	0.09 (0.01) *	0.09 (0.02) *	0.03 (0.00) *	0.04 (0.01) *
High	0.05 (0.02) *	0.06 (0.01) *	0.01 (0.00)	0.01 (0.01) *
**Community illiteracy rate**				
Low	Reference			
Average	-0.07 (0.02) *	-0.06 (0.03) *	-0.00 (0.02)	-0.04 (0.00) *
High	-0.05 (0.02) *	-0.04 (0.03) *	-0.03 (0.02) *	-0.07 (0.01) *
**Community unemployment**				
Low	Reference			
Average	0.02 (0.02)	0.04 (0.03) *	0.01 (0.02)	0.01 (0.00) *
High	-0.06 (0.01) *	-0.03 (0.01) *	-0.02 (0.01) *	-0.02 (0.01) *
**Level 3 (State Level Characteristics)**				
**Rural proportion**				
Low rural proportion	Reference			
Average rural proportion	-0.10 (0.09)	-0.09 (0.02) *	-0.03 (0.01) *	-0.06 (0.06) *
High rural proportion	-0.07 (0.13)	-0.03 (0.01) *	-0.11 (0.05) *	-0.07 (0.02) *
**Health facility per 100,000**				
< 15	Reference			
15 – 25	-0.04 (0.06)	-0.00 (0.01)	0.00 (0.03)	0.06 (0.06) *
> 25	0.04 (0.06)	0.01 (0.02)	0.01 (0.10)	0.11 (0.05) *
**RANDOM EFFECTS**				
**Community Level**				
Standard Deviation (S. E.)	0.23 (0.00)	0.18 (0.02)	0.06 (0.00)	0.07 (0.00)
Variance Partitioning Coefficient	0.10	0.05	0.03	0.01
**State Level**				
Standard Deviation (S. E.)	0.17 (0.00)	0.14 (0.00)	0.12 (0.02)	0.13 (0.01)
Variance Partitioning Coefficient	0.12	0.06	0.04	0.03
**SAMPLE SIZE**				
Individual-level	12,761	12,761	12,761	12,761
Community-level	1,338	1,338	1,338	1,338
State-level	37	37	37	37
**GOODNESS OF FIT**				
-2 Log Likelihood	-31384.02	-30274.39	-27235.46	-27171.47
LOOIC	63586.20	60769.70	54601.60	54464.20
WAIC	64503.70	60995.80	54735.40	54588.00

^*^statistically significant

Overall, maternal age, education and household wealth status, number of antenatal care visits, employment status, religion, delivery in the hospital/health centre, religion, birth order, whether the mothers wanted the child or to delay the pregnancy, postnatal care, who takes decisions about healthcare utilization were significantly associated with the number of vaccines taken – although these variables were not statistically significant for all the models compared. Similarly, community and state-level characteristics were statistically significant for the models.

## Discussion

The study was conducted using the multilevel Bayesian MCMC approach. It allowed the estimation and adjustments of multiple predictors of the number of vaccine uptake by children aged 12–23 months. In particular, a Bayesian hierarchical regression model was employed to account for the inherent spatial (at community- and state- levels) and temporal autocorrelation within the data to estimate the parameters due to the cluster sampling design. The MCMC techniques, which samples successively from a target distribution, were used to draw the posterior samples; specifically, the Metropolis–Hastings updating steps were used.

Comparing the -2 Log Likelihood, LOOIC and WAIC values of the four Bayesian count models, the Zero-Inflated Negative Binomial model had the lowest value for all model fit comparators used, and we adjudged it to be the best Bayesian count model for the data. We found that Poisson, Negative Binomial and Zero-Inflated Poisson models were less appropriate for modelling in the presence of extra zero counts. The study also observed a disparity in mean and variance of the number of vaccine uptake.

Our findings validate the assumption that the Poisson model may not be appropriate to fit the data, as the Poisson regression model performed the worst among the four methods. The data, in the presence of many zeros, is very heavily skewed. Since one of the assumptions of the Poisson regression is that the mean and variance share the same parameter. The Zero-Inflated Poisson model performed better than the Negative Binomial because of the presence of excess zeros in the data. Notably, the Poisson regression model was the least appropriate in the presence of overdispersion and “zero inflation” recorded in this data.

Our finding aligns with existing literature. Studies have reported that the Negative Binomial model has the best performance of count models for count health outcomes, and by extension, the Zero-Inflated Negative Binomial model in the presence of overdispersion and excess zeros and high variability – one of which is vaccine uptake among Children in Nigeria [1–4].

On the factors associated with vaccine uptake, the study corroborates findings from previous literature that identified child-specific, parental and household characteristics as important predictors of child vaccine uptake [9–12]. The study also established that factors that may explain childhood vaccine uptake may vary by the geographical location of the mothers [10, 16].

### Strength and limitations

A major strength of this study was that the study pooled data between 2003 and 2018 in Nigeria to select the best count approach for modelling the number of vaccine uptake among children aged 12–23 months in Nigeria, and applied the Bayesian framework against the traditional -frequentist approach. Nevertheless, Bayesian frameworks are more computationally intensive and thereby impacted the speed of the model despite parallelization. A limitation is that there may have been under-reporting in the number of vaccine uptake; the data collection protocol assumed no vaccination if there is no record on vaccination by the respondent or if the mother cannot remember if the child took the vaccine.

## Conclusion

This study aimed to evaluate the fit and performance of different Bayesian count models. Of the four Bayesian count regression models compared in terms of -2 Log Likelihood, LOOIC and WAIC for modelling the factors associated with the number of vaccine uptake among children aged 12 – 23 months in Nigeria, the Zero-Inflated Negative Binomial model performed most. Data with many zeros are often encountered in public health outcomes. Failure to account for “zero inflation” in the data while analyzing such data may result in inferences that are not true.

Vaccine uptake among children aged 12–23 months also varied across individual-, community-, and state-level characteristics. Specifically, the study observed significant improvements in uptake recorded across the survey years. In summary, this study has enhanced the understanding of the complexities surrounding vaccine uptake among children and provides valuable insights for public health interventions and policies in Nigeria.

## Recommendation

This study characterized the robustness of different count-modelling predictive models to assess their suitability for vaccination uptake in Nigeria. Future research needs to adopt the zero-inflated negative binomial model for studies with high variability and overdispersion to maximize model robustness, hence, enhancing model accuracy and ultimately providing better recommendations for policy and decision-making. We would also recommend that further research be done to explore extensively the factors associated with vaccine uptake among children in Nigeria.

## Data Availability

Data used for this study are available on the DHS website, https://dhsprogram.com

## Abbreviations

-2 Log LL: -2 Log-likelihood
LOOIC: Leave-one-out cross-validation information criterion
WAIC: Watanabe-akaike/widely applicable information criterion
GLM: Generalized linear model
MCMC: Markov chain monte carlo
HMC: Hamiltonian monte carlo
GLMM: Generalized linear mixed model
BCG: Bacillus-calmette-guérin
DPT: Diphtheria-pertussis-tetanus
NDHS: National demographic and health survey
ANC: Antenatal care
